# Individual Skills Based Volunteerism and Life Satisfaction among Healthcare Volunteers in Malaysia: Role of Employer Encouragement, Self-Esteem and Job Performance, A Cross-Sectional Study

**DOI:** 10.1371/journal.pone.0077698

**Published:** 2013-10-23

**Authors:** Chanthiran Veerasamy, Murali Sambasivan, Naresh Kumar

**Affiliations:** 1 Linton University College, Nilai, Malaysia; 2 Taylor's Business School, Taylor's University, Subang Jaya, Malaysia; 3 Global Entrepreneurship Research and Innovation Center (GERIC), Universiti Malaysia Kelantan, Putrajaya, Malaysia; University Hospitals of Geneva, Switzerland

## Abstract

The purpose of this paper is to analyze two important outcomes of individual skills-based volunteerism (ISB-V) among healthcare volunteers in Malaysia. The outcomes are: job performance and life satisfaction. This study has empirically tested the impact of individual dimensions of ISB-V along with their inter-relationships in explaining the life satisfaction and job performance. Besides, the effects of employer encouragement to the volunteers, demographic characteristics of volunteers, and self-esteem of volunteers on job performance and life satisfaction have been studied. The data were collected through a questionnaire distributed to 1000 volunteers of St. John Ambulance in Malaysia. Three hundred and sixty six volunteers responded by giving their feedback. The model was tested using Structural Equation Modeling (SEM). The main results of this study are: (1) Volunteer duration and nature of contact affects life satisfaction, (2) volunteer frequency has impact on volunteer duration, (3) self-esteem of volunteers has significant relationships with volunteer frequency, job performance and life satisfaction, (4) job performance of volunteers affect their life satisfaction and (5) current employment level has significant relationships with duration of volunteering, self esteem, employer encouragement and job performance of volunteers. The model in this study has been able to explain 39% of the variance in life satisfaction and 45% of the variance in job performance. The current study adds significantly to the body of knowledge on healthcare volunteerism.

## Introduction

“*An individual has not started living until he can rise above the narrow confines of his individualistic concerns to the broader concerns of all humanity” – Martin Luther King Jr.*

Volunteerism, an altruistic activity that is intended to improve the quality of human lives, is the practice of an individual or a group of individuals contributing time or skills without the motivation of financial or material gain [1]. Based on the literature, there are different types of volunteering. They are: (1) skills based volunteering (individual-skill based and corporate-skill based), (2) corporate volunteering, (3) volunteering in developing and under-developed countries, (4) environmental volunteering, (5) virtual volunteering, (6) micro-volunteering, (7) volunteering in an emergency, (8) volunteering in schools, (9) community volunteering and (10) international work camps [1]–[6]. In this research, we focus on individual skill-based volunteerism (ISB-V) and volunteers attached to St. John Ambulance Service of Malaysia. Volunteerism (individual and corporate) is becoming popular in Malaysia and recently many NGOs have collaborated and started the “Do Something Good' campaign in Malaysia. The Government of Malaysia has declared 2013 as the National Volunteer Year and a youth volunteer fund to the tune of $33 million has been set up. The fundamental questions addressed in this research are: (1) What is the relationship between the individual dimensions of ISB-V, job performance and life satisfaction? (2) How does employer encouragement and self-esteem of healthcare volunteers affect their job performance and life satisfaction?

According to Points of Light Foundation (2006), the use of personal and professional skills and talents for the benefit of the communities is ISB-V. ISB-V is defined as a “service to nonprofit organizations by individuals or groups that capitalize on personal talents or core business or professional skills, experience or education, often for the purpose of building organizational strength and increasing capacity” (p. 8) [1]. ISB-V can involve three parties: the individuals who are volunteers, non-profit organization or volunteer organization and the communities (nursing homes, children's homes) that receive the volunteer services. Volunteerism benefits both the giver and the recipient and this is especially true with ISB-V [8] and people who volunteer for reasons such as social interaction and self-satisfaction [9]. A few studies have established the link between volunteerism and life satisfaction through enhanced psychological well-being [10]–[12] and this satisfaction results in repeated engagements with volunteering activities [13]. It is difficult to compute volunteerism in a holistic manner and various researchers have measured volunteerism by looking at three distinctive sub-variables, such as the direct and indirect contact [14], volunteer duration and volunteer frequency [15]–[18]. In this research, these three are considered as the dimensions of ISB-V.

Based on the judgment-type theory, life satisfaction of an individual is a function of a comparison between perceived life accomplishments and a set of evoked standards (ideal life-plan) set by the individual without any external imposition [19]. Life satisfaction is a cognitive judgmental process (Frisch, 2000). In this study, life satisfaction is the individual's overall “satisfaction with life as a whole” (p. 8) [20] as opposed to satisfaction with different life domains.

Skills transfer from volunteer activities to paid employment is not a new idea. In fact, the impression of transferring skills gained through volunteer work to paid employment got its first major impetus in 1964 when the United States Employment Center assigned volunteer interviewers to work with professionals to help women find part-time employment opportunities [21]. According to Phillips and Phillips (2000), skills gained through volunteerism can be put to use effectively in job searches. Research on skills transfer from volunteer work to paid employment has been conducted by Hybels (1978) and Schram (1985). They have argued that skills developed through volunteerism improve job opportunities and job performance of volunteers. The studies by Austin (1998) and Ross (1997) corroborate these findings by referring to reports by CEOs of Fortune 500 companies who claim that volunteering has helped them create new contacts and improve their company's reputation in addition to individual fulfillment and skill development. Some of the life and work skills that volunteers develop through volunteerism are group communication, leadership, interpersonal communication social justice, critical thinking, workplace literacy skills and moral character development [9]. Volunteering, in general, enhances the well-being of volunteers and increases the frequency of positive emotions [27]. Many volunteers enhance their career prospects, command higher salaries, and get better jobs because of their work [28]–[30]. In this research, we consider job performance as one of the outcomes of ISB-V.

Employer (paid employment) encouragement to volunteers plays an important role in enhancing the job performance of volunteers through skill development, enhanced morale, increased motivation and loyalty to the organization. Volunteering improves problem-solving abilities and enhances the health of volunteers by reducing their stress levels, retaining their mental acuity and heightening their sense of self-worth and self-esteem [11]. In this research, we consider the role of employer encouragement in enhancing the job performance and life satisfaction of volunteers.

Many researchers have been unanimous in accepting the fact that volunteering, among other things, enhances the self-esteem of the volunteer [11], [31]. According to Judge and Bono (2001), self-esteem is one of the four best predictors of job performance. Self-esteem and life satisfaction of individuals are highly correlated [33]. In this research, we consider the role of self-esteem of volunteers in improving their job performance and life satisfaction.

The relationship between job performance and life satisfaction has been subjected to a considerable debate by researchers. Some researchers have argued the influence of job performance on life satisfaction and some have argued the opposite [34]–[37]. In this research, we argue the influence of job performance enhanced by volunteering activities, employer encouragement and self-esteem on life satisfaction.

The contributions of this study will be threefold. First, there is a dearth of studies concerning ISB-V and its outcomes [1], [10], [12]. In this study, we will consider the effect of individual dimensions of ISB-V on job performance and life satisfaction. The inter-relationships between the dimensions of ISB-V will also be considered in this research. Second, we will additionally consider the roles of employer encouragement, self-esteem and demographic variables on job performance and life satisfaction of volunteers. We will test the integrated model using Structural Equation Modeling (SEM). Third, the study will be conducted among the healthcare volunteers in Malaysia. Studies from this part of the world on volunteerism are a rarity.

### Theoretical framework and hypotheses development

**Figure 1:**
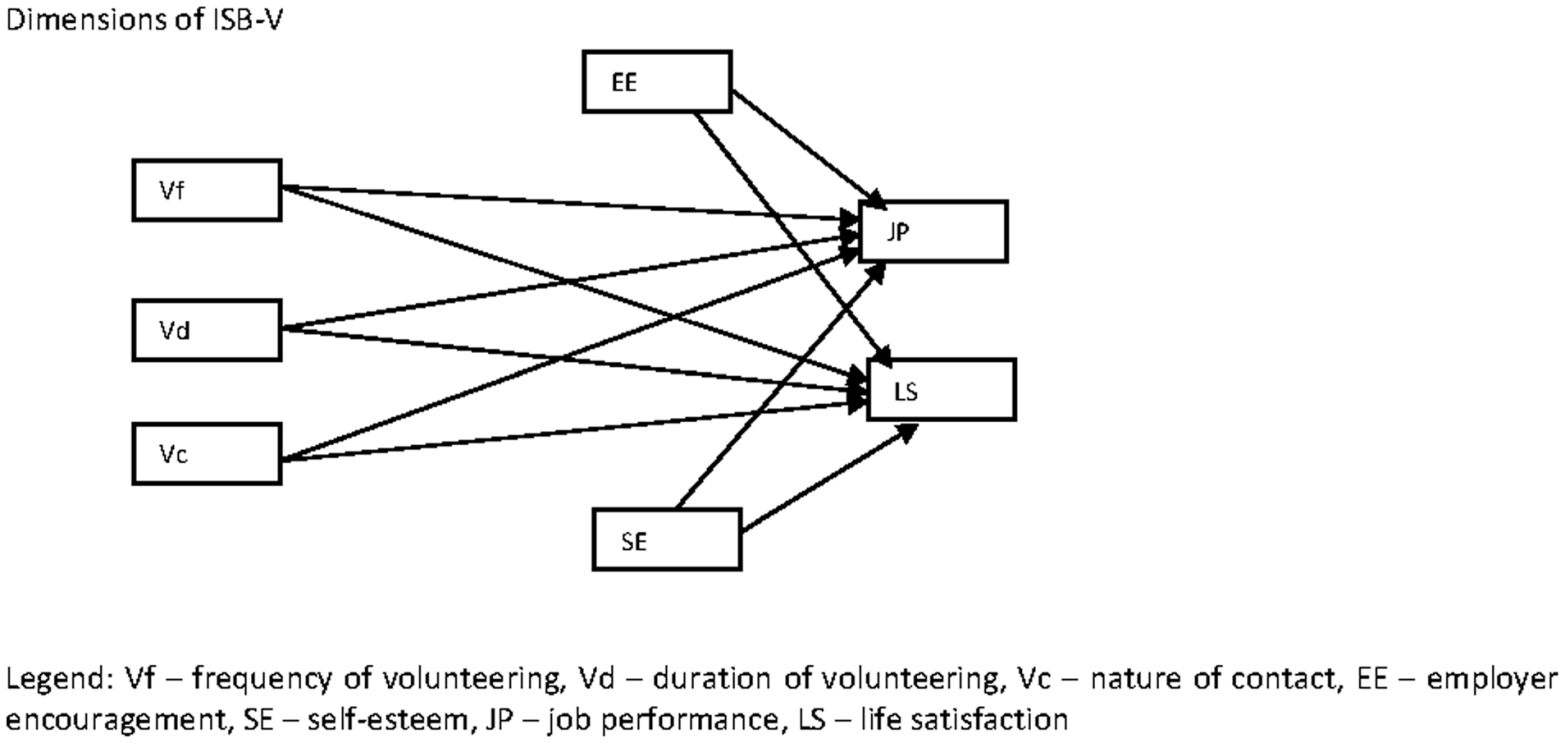
Initial theoretical framework.

Many theories have been developed to explain volunteerism from different perspectives: (1) Volunteer Process Model [31], [37], [39]; (2) the Role Identity Theory [40]; (3) the Human Capital Theory [41]; (4) the Functional Theory [42]; (5) the Four Motive Theory [43]; (6) the Utility Theory [44]; (7) the Theory of Altruism [45]; (8) the Socialization Theory [41]; and (9) Social Exchange Theory [46]. While other theories focus on explaining why people volunteer, role identity theory goes a step further and explains why people continue to volunteer. For research on sustained volunteerism, this theory is more relevant. The Role Identity theory of behavior hypothesizes that people develop a role identity as a particular type of actor as they repeatedly engage (sustained volunteerism) in a type of activity [40]. A person who volunteers more than a few times may come to believe that he/she is the kind of person who volunteers and eventually concludes that volunteering is a vital part of who he/she is. Sustained volunteerism results in better mental well-being such as reduced stress and depression levels, enhanced self-esteem, improved job performance and overall life satisfaction of volunteers [11], [12], [47], [48]. Figure 1 shows the framework used in this study.

### ISB-V and life satisfaction of volunteers

Initially, the explanation to life satisfaction of volunteers came from the Social Interest theory [49]. This is rooted in the fact that volunteerism is a social support phenomenon which is a component of social interest domain. Social interest involves a sense of belongingness and probably serves as the strongest explanatory mechanism for social integration experienced when a person engages in volunteering acts. Social interest has been shown to be an important contributor to one's satisfaction with life, and is considered a key component of positive mental health [49]. Life satisfaction and social interest are believed to share a reciprocal relationship, where individuals who have more aptitude for establishing social ties with others tend to be well liked and more accepted than those with lower levels of this ability [50]. As indicated earlier, continuous engagement in acts of volunteerism is better explained by Role Identity theory. Initial engagement in volunteerism is probably due to social interest and social integration. For an individual to sustain that interest for a longer duration, he/she must identify the self with the act and believe that volunteerism is a vital part of who he/she is. Since there are a few studies that have established a strong link between volunteerism and life satisfaction [31], [51], we hypothesize as follows:

*H1: There is a positive relationship between ISB-V (frequency of volunteering, duration of volunteering and the type of contact – direct and indirect) and life satisfaction of volunteers.*

### ISB-V and job performance of volunteers

Volunteering sends positive signals in the labor market. Thus, many volunteers enhance their career prospects, command higher salaries, and get better jobs because of their work [28]–[30]. A theoretical mechanism that could be at work for the explanation of the relationship between volunteerism and job performance is the Work-Family Theory. Volunteering is a non-work activity that can both enrich and detract from regular employment attitudes and behaviors [52].

Although work-family researchers initially focused almost exclusively on the conflicting nature of work and non-work activities, these activities are inextricably linked and have significant repercussions on each other [53]. These researchers quickly noted the possibility that work and non-work activities can also be mutually beneficial [52], [53]. Since then, researchers have found evidence that work and non-work relationships can be concurrently beneficial and unfavorable [54], [55]. A majority of volunteering and regular employment studies provides qualitative evidence that individuals develop skills, such as teamwork and leadership skills, through volunteering that ultimately benefit their employers [56], [57]. Booth et al., (2009) have empirically demonstrated that volunteering is positively related to acquiring skills that are beneficial at the workplace. A series of interviews with Fortune 500 CEOs have highlighted the fact that volunteering helps create new social contacts that can benefit regular employment [25], [26]. Based on these arguments, we posit the following hypothesis:

*H2: There is a positive relationship between ISB-V (frequency of volunteering, duration of volunteering and the type of contact – direct and indirect) and job performance of volunteers.*

### Job performance and life satisfaction of volunteers

The relationship between job satisfaction and life satisfaction has been studied by many researchers [58]–[60]. Comparatively, there are a fewer studies linking job performance and life satisfaction [34]–[36]. These studies suggest that it is possible to have a two-way relationship between job performance and life satisfaction. In this study, we argue that volunteerism improves work related skills that can be extremely beneficial to the individual (who is a volunteer) and the employer [56], [57]. This improvement in job performance of the volunteers can lead to their increased life satisfaction. According to Johnson et al. (2008), life satisfaction of an individual depends upon that individual's perception regarding the ‘meaningfulness’ of his/her life. They have argued that personal accomplishments (better job performance) achieved through paid employment can lead to a sense of satisfaction with life. Based on these arguments, we hypothesize as follows:

*H3: There is a positive relationship between job performance and life satisfaction of volunteers.*

### Employer encouragement and volunteerism

The Perceived Organizational Support Theory (POS) offers a theoretical mechanism that could be at work for explaining the relationship between employer encouragement to volunteer and job performance and life satisfaction of volunteers. POS is an individual's belief of the care and concern that his/her organization has for his/her wellbeing and performance or contribution [61], [62]. Based on POS, individuals who perceive that their organization supports them will naturally respond positively. In effect, the individuals and the organization enter a psychological contract where the employees (who are volunteers) exchange their positive organizational behavior, such as engagement and performance, for the organization's support [63]. According to Grant (2012), employees view their employers as more caring and pro social when they provide support to volunteering activities. The perception of support received from the organization creates a sense of obligation to the organization and this yields positive employee practices such as organizational attachment [61], organizational citizenship behavior [64] and affective commitment [65]. These positive attitudes and behaviors result in increased commitment toward the organization's performance [62], [66] and therefore, better job performance. Volunteers who get support from the employers are highly satisfied and committed to their jobs. Higher satisfaction and performance in their jobs lead to greater life satisfaction of volunteers [35]. Based on the above arguments, we posit the following hypotheses:

*H4: There is a positive relationship between employer encouragement and job performance of volunteers.*

*H5: There is a positive relationship between employer encouragement and life satisfaction of volunteers.*

### Self-esteem and volunteerism

Self-esteem, as measured by the Rosenberg Self-Esteem Scale [67], refers to an individual's favorable or unfavorable view of one's self — an appraisal of one's self [68]. Individuals with high self-esteem tend to evaluate themselves positively, both cognitively and affectively [69]. They also tend to respect themselves and consider themselves worthy [68]. Several longitudinal studies suggest that volunteers enjoy increased self-efficacy, self-esteem, and other positive affects that result in lowered risk of morbidity among the volunteers [12], [69]. The impact of self-esteem on job performance and life satisfaction is well established in literature [66], [70]. Based on the above arguments, we posit the following hypotheses:

*H6: There is a positive relationship between self-esteem and job performance of volunteers.*

*H7: There is a positive relationship between self-esteem and life satisfaction of volunteers.*

## Methods

### Sample and Sampling Procedures

St. John Ambulance Malaysia (SJAM) was established in 1908 [71]. SJAM has more than 60,000 volunteers nationwide and these volunteers dedicate 3.6 million man-hours annually for the service of humankind. SJAM provides services such as haemodialysis centers, 24-hour emergency and non-emergency ambulance services, home nursing care for the invalid and other humanitarian services. The sampling technique adopted was stratified random sampling (each strata – each state of Malaysia). There are 14 states and federal territories in Malaysia and the sample size was divided proportionately. The approval to conduct the study was obtained from the headquarters of St. John Ambulance, Malaysia.

Data were gathered through questionnaires administered through the person in-charge of volunteers at each state. The questionnaire consisted of separate sections for each construct: ISB-V, employer encouragement, self-esteem, job performance and life satisfaction. The job performance construct did not capture the performance in the volunteering activity. It captured the performance in the full time or part time paid jobs that volunteers performed in addition to volunteering work. Besides these constructs, demographic information such as age, race, sex, education and full-time or part-time job information was also captured. Permission to use the items under each construct was obtained from each author who developed the items. The final questionnaire was vetted by the faculty committee (supervisory).

Out of 1000 questionnaires sent, we received responses from 366 volunteers (response rate – 36.6%). The responses received from different regions of Malaysia were as follows: North – 33.3%, Central – 20.8%, East – 11.7%, South – 21.1% and Sabah and Sarawak – 13.1%. The characteristics of the respondents were: (1) About 60% were males, (2) one third of respondents were less than 25 years of age and about 22% of the respondents were more than 46 years, (3) about 80% of the respondents were diploma holders or had higher qualifications, (4) all the respondents were working and 87% were employed full time and the remaining part time, (5) about 48% of the respondents were holding managerial positions, (6) on an average, respondents had more than 14 years of volunteering experience and had contributed more than 14 hours per month for volunteering activities and (7) about 64% of respondents were involved in ‘direct contact’ volunteering as opposed to ‘indirect contact’ volunteering.

### Measures

#### Dependent variables: Life satisfaction (LS) measure

The volunteer's life satisfaction was measured by using the five-item scale of “Satisfaction with Life Scale” which was developed by Diener et al. (1985). According to Diener et al. (1985), the Satisfaction with Life Scale (SWLS) had been heavily used as a measure of the life satisfaction. The recorded reliability score for this scale was between 0.82 and 0.87 and for study was 0.86. The items in this scale were: “The conditions of my life are excellent”; “In most ways my life is close to my ideal”; “So far I have gotten the important things I want in life”; “I am satisfied with my life”; and “If I could live my life over, I would change almost nothing.” All items used a response scale ranging from 1 =  Strongly Disagree to 5 =  Strongly Agree. A Confirmatory Factor Analysis (CFA) was conducted and the fitness statistics were: Ch-square  = 2.002, degrees of freedom (df)  = 4, Root Mean Square Error Approximation (RMSEA)  = 0.000, Root Mean Square Residual (RMR)  = 0.008 and Normed Fit Index (NFI)  = 0.99.

#### Job performance (JP) measure

There were three components of job performance: the focal performance, contextual performance, and withdrawal behavior. Focal performance in this study was defined as the degree to which a volunteer meets or exceeds expectations about task role requirements [13], [76], [77]. Focal performance was operationalized as a perception of task proficiency and role dedication using the 8-item scale developed by Hainsworth and Barlow (2001). A recent study by Cromer (2009) recorded a reliability score of 0.84. The items in this scale were: “I work efficiently”; “I understand most work procedures”; “I learn new job skills quickly”; “I am technically proficient in my job”; “I take on new assignments with enthusiasm”; “I persist in overcoming obstacles to complete a task”; “I solve most work-related problems without help”; and “I take responsibility for work-related failures”. All items used a response scale ranging from 1 =  Strongly Disagree to 5 =  Strongly Agree. A CFA was conducted and the fit statistics were: Chi-square  = 22.683, df  = 15, RMSEA  = 0.037, RMR = 0.026, and NFI = 0.99.

Contextual performance in this study is defined as an extra-role, discretionary behavior by volunteers in their workplace that are not formally a part of the specific focal role, directed at support for the organization or helping others [13], [76]. The contextual performance in this study was measured by the volunteers themselves using the eight items from the *Organizational Citizenship Behavior Questionnaire* which was modified by Cromer (2009) and he recorded a reliability of 0.81. The items in this scale were: “I help others who have been absent”; “I volunteer for things that are not required”; “I help orient new people even though it is not required”; “I help others who have heavy workloads”; “I give advance notice if unable to come to work when expected”; “I assist supervisor with his or her work”; “I make innovative suggestions to improve the department”; and “I attend functions not required but that helps organization's image”. All items used a response scale ranging from 1 =  Strongly Disagree to 5 =  Strongly Agree. A CFA was conducted and the fit statistics were: Chi-square  = 25.640, df  = 11, RMSEA  = 0.060, RMR = 0.035, and NFI = 0.98.

In this study, the withdrawal behavior is defined as disengagement behaviors by volunteers at their workplace indicated by discretionary lateness, absenteeism, or turnover [13], [77]. In this study, eight items were taken from the items used by Cromer (2009) and the recorded reliability score is 0.70. The items in this scale were: “I am punctual”; “I take underserved breaks”; “My attendance at the work is above the norm”; “I relax towards the end of the day”; “I spend a great deal of working time in personal phone conversations”; “I do not take unnecessary time off from my work”; “I do not take extra breaks while working”; and “I do not spent time in idle conversation”. All items used a response scale ranging from 1 =  Strongly Disagree to 5 =  Strongly Agree. A CFA was conducted and items that had a factor loading of less than 0.5 were removed. Four items were removed from the withdrawal behavior dimension of job performance and the items were: “I take undeserved breaks”; “My attendance is above the norm in my organization”; “I relax towards the end of the day”; and “I spend a great deal of work time in personal phone conversations”. The fit statistics were: Chi-square  = 0.881, df  = 2, RMSEA  = 0.001, RMR = 0.009, and NFI = 0.99. The overall reliability score of the construct job performance was 0.91.

#### Independent variables: ISB-V measure

ISB-V was measured by adopting the scale from the Giving and Volunteering Survey Instrument published by Independent Sector (1999) and from the national volunteerism survey sponsored by Spirituality and Health [73]. Specifically, ISB-V was measured by frequency (*Vf*), duration (*Vd*) and nature of volunteer contact (direct vs indirect) (*Vc*). Frequency of volunteer service was assessed with one open-ended question: “In the past year, how many hours per month have you spent doing volunteer work?” Even though there are different conceptualizations, the most widespread one is the number of hours spent doing volunteer work that is a single-item reflective measure. Duration of volunteer service was assessed with one question: In total, how many years (or month if less than one year) have you been doing volunteer work? Nature of contact (Direct versus indirect volunteer contact) was assessed with one question: “If you volunteered in some capacity over the past years, what was the nature of the work performed most frequently?” (coded as ‘0’ — indirect contact with people (e.g. fundraising, administrative or clerical work) and coded as ‘1’— Direct contact with people (e.g., giving advise/consultation, information or counseling such as working hotlines, mentoring and training, help line, or visiting people – nursing homes and children's homes, providing companionship, hospital duty, a care facility-Dialysis Centre, providing ambulance service)). Each dimension of ISB-V was assessed with one direct question. In this research, direct contacts are defined as those that involve volunteers providing front line activities with the communities and clients they serve and the indirect contacts are those that assist/support front line activities [7].

#### Employer encouragement (EE) measure

In this study, the employer's encouragement was measured by a five-item scale that was adapted from Zhou and George (2001). This measure was later used by Rodell (2010) and recorded a reliability score of 0.90 and Zhou and George (2001) had recorded 0.73 using this scale. The reliability score in our study was 0.88. The items in this scale were: “Volunteering is encouraged by my employer”; “Participation in volunteer activities is respected by the leadership where I work”; “My employer endorses volunteer opportunities”; “My employer supports involvement in volunteer activities”; “My employer's reward system encourages volunteering”. All items used a response scale ranging from 1 =  Strongly Disagree to 5 =  Strongly Agree. A CFA was conducted and the fit statistics were: Chi-square  = 2.890, df  = 4, RMSEA  = 0.001, RMR = 0.021, and NFI = 0.98.

#### Self-esteem (SE) measure

Self-esteem was measured by a ten-item scale which was developed by Rosenberg (1965). In one of the current research papers, Rodell (2010) recorded a reliability score of 0.84 for self-esteem. The reliability score for this construct in our study was 0.84. The items in this scale were: “I feel that I have a number of good qualities,”; “I feel that I am a person of worth, at least on an equal basis with others,”; “I am able to do things as well as most other people,”; “All in all, I am inclined to feel that I am a failure” (reverse-worded); “I feel I do not have much to be proud of” (reverse-worded); “On the whole, I am satisfied with myself,”; “I have a positive attitude toward myself,”; “I certainly feel useless at times” (reverse-worded); “I wish I could have more respect for myself” (reverse-worded); and “At times I think I am no good at all” (reverse-worded). All items used a response scale ranging from 1 =  Strongly Disagree to 5 =  Strongly Agree. A CFA was conducted and five items that had a factor leading of less than 0.5 were removed from self esteem and the items were: “All in all, I am inclined to feel that I am a failure”; “I feel I do not have much to be proud of”; “I wish I could have more respect for myself”; “I certainly feel useless at times”; and “At times I think I am no good at all”. The fit statistics were: Chi-square  = 3.609, df  = 4, RMSEA  = 0.002, RMR = 0.0152, and NFI = 0.99.

#### Demographic variables measure

Demographic variables used in this research were: age, education level, marital status, gender, current employment (paid) level, nature of industry currently employed in and income level. The variables that require further clarification are current employment level and nature of industry currently employed in. We considered two categories for the current employment level — managerial (coded as 1) and non-managerial (coded as 0) and five categories for the nature of industry currently employed in — manufacturing (coded as 1), banking (coded as 2), healthcare (coded as 3), training and education (coded as 4) and others (coded as 5). However, while using this variable in the SEM model four dummy-coded variables were created. The complete data set can be made available by authors upon request.

### Data Analysis

The data analysis was performed using SPSS and Lisrel 9.1 (student version). SPSS was used for generating descriptive statistics and bivariate statistics between different constructs. Descriptive statistics were generated to answer two questions about Malaysian healthcare volunteers: (1) On an average, how many hours per month and how many years of volunteering were undertaken? (2) What were the ‘levels’ of life satisfaction, job performance, self esteem and employer encouragement experienced? Mean, standard deviation and proportion were used as measures of descriptive statistics.

Bivariate statistics using Pearson correlation were generated to understand the inter-relationships between the constructs. This statistics helped us identify additional relationships especially, with demographic variables that were not hypothesized. In summary, SEM model included both the hypothesized relationships and additional relationships identified through bivariate statistics.

SEM model with the entire hypothesized and additional relationships revealed through bivariate statistics was run using Lisrel 9.1 student version. According to Hair et al. (2010), “SEM is a family of statistical models that seek to explain the relationships among multiple variables. In doing so, it examines the structure of interrelationships expressed in a series of equations, similar to a series of multiple regression equations. These equations depict all the relationships between the dependent and independent variables involved in the analysis.” (p. 711). Since our framework involved multiple relationships between independent and dependent constructs, SEM was the most appropriate tool to use. Our model had the following variables: frequency of volunteering, duration of volunteering, nature of contact during volunteering, self esteem, employer encouragement, job performance, life satisfaction and the demographic variables (gender, age, nature of industry and current employment level).

## Results

### Descriptive statistics

The descriptive statistics of various constructs are given in Table 1. Some mean values deserve mention. The average time spent by volunteers per month is 14.36 hours (standard deviation (sd)  = 8.6) and the average number of years spent by volunteers in volunteering is 14.34 years (sd = 6.94). About 64% of the volunteers were engaged in direct contact activities. The respondents scored moderate on self-esteem (mean  = 3.85, sd = 0.596), job performance (mean  = 3.71, sd  = 0.456) and life satisfaction (mean  = 3.61, sd  = 0.778). Overall, it can be said that sampled volunteers of St. John Ambulance in Malaysia were experienced, were investing significant time on volunteering activities and had ‘moderate’ levels of self-esteem, job performance and life satisfaction.

**Table 1:** Descriptive statistics.

Variables	Levels	Mean	SD
Frequency (Vf) (hours/month)	Moderate	14.36	8.60
Duration (Vd) (years)	Moderate	14.34	6.94
Contacts (Vc)			
Direct	High	64%	
Indirect	Low	36%	
Self-Esteem (SE)	Moderate	3.85	0.596
Job Performance (JP)	Moderate	3.71	0.456
Life Satisfaction (LS)	Moderate	3.61	0.778
Employer encouragement (EE)	Moderate	3.43	0.850

### Bivariate Statistics

In this research, the demographic variables used were: gender, age, education level, marital status, current employment (paid) level, nature of industry currently employed in and income level. We ran a bivariate correlation between the demographic and the other constructs used in this research and the results are given in Table 2. Based on the results, among the demographic variables gender, age, nature of industry and current employment level had significant relationships with duration of volunteering, frequency of volunteering, nature of contact, self esteem, employer encouragement, job performance and life satisfaction.

**Table 2:** Bivariate statistics.

	1	2	3	4	5	6	7	8	9	10	11	12	13	14
1	1.00													
2	−0.20*	1.00												
3	−0.06	0.00	1.00											
4	−0.18*	0.67*	0.02	1.00										
5	−0.05	0.09	−0.03	0.06	1.00									
6	−0.14*	0.24*	0.14*	0.25*	−0.13**	1.00								
7	−0.22*	0.34*	0.36*	0.34*	0.01	0.32*	1.00							
8	−0.07	0.03	0.02	0.01	0.12**	0.07	0.02	1.00						
9	0.01	0.13**	0.01	0.10	0.04	0.18*	0.03	0.64*	1.00					
10	−0.01	0.01	0.02	−0.04	0.16*	−0.11**	0.02	0.06	−0.00	1.00				
11	−0.15*	0.11**	0.04	0.02	0.16*	0.20	0.07	0.24*	0.19*	−0.02	1.00			
12	0.04	−0.10	−0.05	0.01	0.01	0.13**	−0.10	0.11**	0.12**	−0.02	0.24*	1.00		
13	−0.07	0.09	0.00	0.06	0.19*	0.25*	0.06	0.21*	0.20*	−0.05	0.65*	0.22*	1.00	
14	−0.03	0.04	−0.01	0.07	0.07	0.24*	0.02	0.17*	0.22*	−0.16**	0.58*	0.16*	0.49*	1.00

(*— significant at 0.01 level of significance, ** — significant at 0.05 level of significance).

Legend: 1 – Gender, 2 – Age, 3 – Educational level, 4 – Marital status, 5 – Nature of industry, 6 – Current employment level, 7 – Income level, 8 – Frequency of volunteering, 9 – Duration of volunteering, 10 – Nature of contact, 11 – Self esteem, 12 – Employer encouragement, 13 – Job performance, 14 – Life satisfaction.

### Testing of hypotheses

In this research, ISB-V (Vd, Vf and Vc), self-esteem, employer encouragement, job performance and the demographic variables (gender, age, nature of industry and current employment level) were the independent constructs and life satisfaction was the dependent construct. The fit statistics of the SEM model are: Chi-square  = 29.96 (degrees of freedom  = 28), chi-square/degrees of freedom  = 1.07 (threshold value — <3), p-value  = 0.36 (threshold value - >0.05), RMSEA (Root mean square error approximation)  = 0.012 (threshold value — <0.08), NFI (Normed-fit index)  = 0.97 (threshold value — >0.9), RMR (Root mean square error residual)  = 0.033 (threshold value — <0.08). Based on the results, the model fit is good. Thirty nine percent of the variation in life satisfaction (R2 = 0.39) has been explained by ISB-V, self-esteem, employer encouragement, job performance and current employment level of volunteers. These constructs seem to predict a significant portion of life satisfaction among healthcare volunteers (St. John Ambulance) in Malaysia.

**Figure 2:**
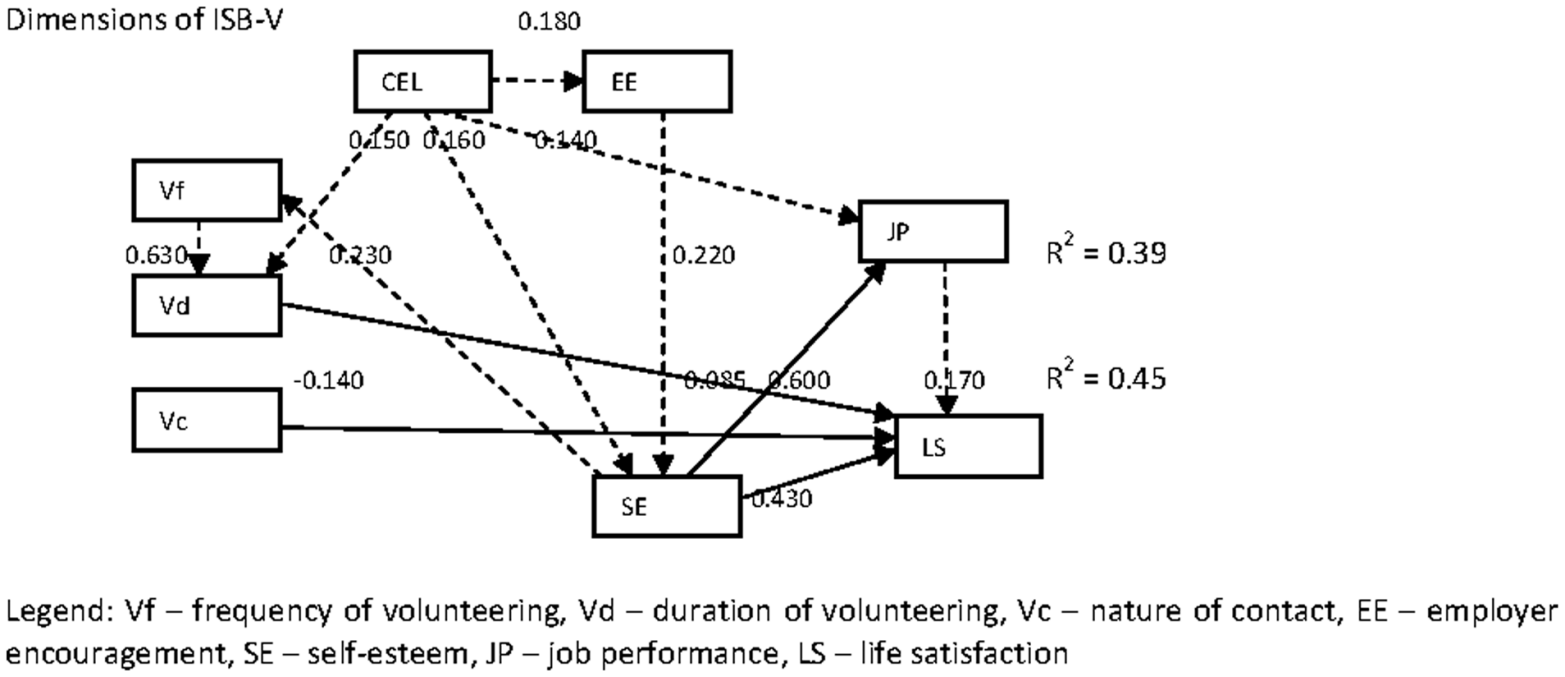
Final framework (based on significant coefficients).

The next question is: Which are the constructs that have significant relationships with life satisfaction of volunteers? Table 3 gives the coefficients of each variable. The final framework (including, inter-relationships between independent variables) with the significant coefficient values are given in Figure 2. The following inferences can be drawn from the SEM output.

**Table 3:** Coefficient values of various paths.

SNo	Path	Coefficient	t-value	p-value
1	Vf → JP	0.020	0.380	0.354
2	Vd → JP	0.051	1.000	0.165
3	Vc → JP	−0.046	−1.170	0.129
4	EE → JP	0.057	1.410	0.088
**5**	**SE → JP**	**0.600**	**14.970**	**0.000**
6	Vf → LS	−0.046	−0.860	0.200
**7**	**Vd → LS**	**0.085**	**2.020**	**0.029**
**8**	**Vc → LS**	**−0.140**	**−3.290**	**0.002**
**9**	**JP → LS**	**0.170**	**3.100**	**0.003**
**10**	**SE → LS**	**0.430**	**8.020**	**0.000**
11	EE → LS	−0.003	−0.069	0.473
12	EE → Vf	−0.019	−0.220	0.414
**13**	**SE → Vf**	**0.23**	**4.410**	**0.000**
14	EE → Vd, Vc	Model fit not good		
15	SE → Vd, Vc	Model fit not good		
**16**	**Vf → Vd**	**0.630**	**15.860**	**0.000**
17	Vc → Vf, Vd	Model fit not good		
18	**CEL → Vd**	**0.15**	**3.770**	**0.002**
19	**GND → SE**	**−0.11**	**−2.260**	**0.036**
20	**CEL → SE**	**0.16**	**3.100**	**0.006**
21	**CEL→ EE**	**0.13**	**2.510**	**0.022**
22	**CEL → JP**	**0.14**	**3.530**	**0.002**
23	AG and NI	Was not significant with other variables		

Legend: Vf – frequency of volunteering, Vd – duration of volunteering, Vc – volunteering nature of contact, EE – Employer encouragement, SE – Self esteem, JP – Job performance, LS – Life satisfaction, GND – Gender, CEL – Current employment level, AG – Age, NI – Nature of industry.

(Highlighted values significant at 0.05 level of significance).

First, the hypothesis that tests the relationship between ISB-V and life satisfaction is partially supported. Among the three dimensions of ISB-V, duration of volunteering (*β = 0.085, t-value  = 2.020, p-value  = 0.029*) and nature of contact (*β = −0.140, t-value  = −3.290, p-value  = 0.002*) have a significant impact on life satisfaction. A few studies have established a strong link between (general) volunteerism and life satisfaction [31], [51]. In our research, we have specifically tested ISB-V. The results indicate that (1) life satisfaction is more for volunteers who have been volunteering for a long time and (2) indirect contact results in higher life satisfaction. In order to further analyze the effect of nature of contact on life satisfaction, we ran an independent ‘t’ test. Based on the results of the test (*t-value  = 3.328, p-value  = 0.001*), mean life satisfaction level with indirect contact is 3.78 (sd  = 0.666) and with direct contact is 3.52 (sd  = 0.822). The frequency of volunteering is significantly related to the duration of volunteering (*β = 0.630, t-value  = 15.860, p-value  = 0.000*). This result suggests that the sampled volunteers who have been volunteering for a longer duration of time spend more hours volunteering every month.

Second, the hypothesis that addresses the relationship between ISB-V and job performance is not supported. A few researchers have found proof that work and non-work relationships can be concurrently beneficial and unfavorable [54], [55] and in general, the research linking volunteerism and job performance is largely inconclusive. In our research, it has turned out that there is no significant effect of ISB-V on job performance.

Third, the hypothesis that argues the relationship between job performance and life satisfaction is supported (*β = 0.170, t-value  = 3.100, p-value  = 0.003*). Our result supports the conclusion of a few recent studies [34]–[36].

Fourth, the hypotheses that test the role of employer encouragement on job performance and life satisfaction are not supported. Our results contradict the findings of Saks (2006) and Rodell (2010). We also tested (not hypothesized) the relationship between employer encouragement and ISB-V. The result shows that the relationship between them is insignificant. However, the relationship (not hypothesized) between employer encouragement and self-esteem is supported (*β = 0.220, t-value  = 4.520, p-value  = 0.000*).

Fifth, the hypotheses that test the relationships between self-esteem and job performance (*β = 0.600, t-value  = 14.970, p-value  = 0.000*) and between self-esteem and life satisfaction (*β = 0.430, t-value  = 8.020, p-value  = 0.000*) are supported. The impact of self-esteem on job performance and life satisfaction is well established in literature [66], [70] and our results support the findings. Even though it has not been hypothesized, we have studied the relationship between self-esteem and ISB-V. The result indicates that self-esteem has a significant relationship with frequency of volunteering (*β = 0.230, t-value  = 4.410, p-value  = 0.000*) and it suggests that sampled volunteers who are high on self-esteem tend to spend more hours per month on volunteering.

Sixth, the effects of demographic variables on other constructs are revealing. Among the seven variables, four variables (age, gender, nature of industry, and current employment level) have been used in the SEM model based on the results of bivariate statistics. Of the four variables, current employment level and gender have shown evidence of some relationships. The current employment level has significant positive relationship with duration of volunteering (*β = 0.15, t-value  = 3.77, p-value  = 0.002*), self esteem (*β = 0.16, t-value  = 3.100, p-value  = 0.006*), employer encouragement (*β = 0.13, t-value  = 2.510, p-value  = 0.022*) and job performance (*β = 0.14, t-value  = 3.530, p-value  = 0.002*). These results imply that Malaysian healthcare volunteers of St. John Ambulance who are employed as managers in their paid employment spend more time on volunteering, have higher self-esteem, get more encouragement from their employers and have better job performance when compared to volunteers who perform non-managerial jobs. The gender has significant relationship with self esteem of volunteers (*β = 0.11, t-value  = 2.26, p-value  = 0.032*) indicating that male volunteers have higher levels of self esteem.

## Discussion

This research set out to answer two fundamental questions: (1) What is the relationship between the individual dimensions of ISB-V, job performance and life satisfaction? (2) How does employer encouragement and self-esteem of healthcare volunteers affect their job performance and life satisfaction? Our study has also addressed an auxiliary question: What are the effects of demographic characteristics of healthcare volunteers on ISB-V, self esteem, employer encouragement, job performance and life satisfaction? In the process of answering these questions, the framework developed has been able to address 39% of the variance in life satisfaction and 45% of the variance in job performance of sampled healthcare volunteers who offer their services to St. John Ambulance. A few studies have analyzed skills-based volunteerism among older adults [8], [51], [79]. In our study, most of the volunteers are young (78% less than 46 years of age with a one-third less than 25 years) and our study unlike others has looked at the inter-relationships between the dimensions of the independent constructs. An interesting result of our study is that in spite of higher percentage of young volunteers, age does not have affect on any of the constructs.

Among the dimensions of ISB-V, duration of volunteering and nature of contact have direct relationships with life satisfaction and frequency of volunteering has an indirect effect on life satisfaction through duration of volunteering. Volunteerism enhances life satisfaction through increased psychological well-being [10]–[12] and this satisfaction, in turn, results in repeated engagements with volunteering activities [13]. Based on the role identity theory, sustained volunteerism results in better mental well-being such as reduced stress and depression levels, enhanced self-esteem, improved job performance and overall life satisfaction of volunteers [11], [12], [47], [48]. Therefore, ISB-V has a significant positive effect on the life of healthcare volunteers.

The effect of ISB-V on job performance has been disappointing. None of the dimensions of ISB-V has an impact on job performance. There are two possible explanations for this result. First, since the job performance measured in this research is self-reported, a plausible explanation is that the majority of healthcare volunteers of St. John Ambulance, Malaysia may not see the benefits their voluntary activities have on their jobs (paid). Second, the jobs performed by volunteers may not be aligned with the skills acquired by the volunteers. According to Points of Light Foundation (2006), only a small percentage of the organizations align roles with skill acquired during volunteering. Based on the literature review, it can be said that the results from the studies linking volunteerism and job performance of volunteers have been inconclusive [54], [55]. However, our study shows that how well the volunteers perform in their (paid) jobs has a significant impact on their life satisfaction. As an answer to the first question we have raised earlier, ISB-V does have an impact on life satisfaction but not on job performance. What are the implications?

Theoretically, our study lends a strong support to the influence of ISB-V on life satisfaction. This is good news for individuals who desire to volunteer (skill-based) and who are already volunteering. In our sample, the average duration of volunteering is 14.34 years (sd  = 6.94 years). It is obvious that the volunteers have repeatedly engaged in volunteering activities. The role identity theory may partially explain the continuous engagement of volunteers due to higher levels of life satisfaction.

An interesting result in our study is the (absence of the) direct role of employer encouragement on job performance and life satisfaction of volunteers. A few researchers have argued that the employers by allowing the employees to contribute to volunteering are increasing their commitment, performance and satisfaction levels [11], [12], [47], [48]. According to Volunteer Canada, companies in Canada are increasingly providing more support to their employees to indulge in volunteering. Imagine Canada conducted a national survey in 2006 and found that more than 35% of the companies in Canada encourage or accommodate employee volunteer activities during working hours. However, in Malaysia the role of employer encouragement is not high as compared to countries like Canada. According to The Star (2013), a few companies in Malaysia have started to support the employees who perform volunteering activities and it may take a few years for more companies to support employee volunteering.

Our study has addressed the role of demographic characteristics of volunteers. Of all the characteristics, current employment level (managerial or non-managerial) has positive impact on duration of volunteering, self esteem, employer encouragement and job performance of healthcare volunteers in Malaysia. Specifically, the result shows that volunteers employed as managers in their paid employment derive maximum benefits through volunteering.

Volunteers develop a high level of self-esteem by continuously engaging in volunteering activities and our study shows that male volunteers have higher levels of self-esteem than female volunteers. The self-esteem, in turn, has a positive influence on job performance and life satisfaction [66], [70]. Therefore, the employers by motivating and by providing physical, moral and financial support to the employees to engage in volunteering are increasing their loyalty and commitment towards the organization. Even though, our study has indicated that employer encouragement is not high in Malaysia, we strongly argue in favor of organizations that recognize the volunteering activities of their employees in a positive manner. Our study has also shown that higher self-esteem of volunteers motivates them to indulge more frequently in volunteering and this indirectly increases life satisfaction. Our results indicate that the effects of all variables converge on life satisfaction of volunteers.

There are many non-governmental organizations (NGOs) in Malaysia that offer healthcare services. Some of them are: Diabetes Society of Malaysia, Epilepsy Society of Malaysia, Mercy Malaysia, Malaysian Mental Health Association and Hospis Malaysia. These NGOs depend on the volunteers who possess special skills in handling healthcare services. We believe that the results of our study can be generalized to include the volunteers attached to these NGOs. It is intriguing to note in our research that employer support did not have a significant role to play in the job performance and life satisfaction of sampled volunteers. However, we believe that increased employer support can help in improving the job performance and life satisfaction of volunteers which in turn can encourage more and more citizens to take up volunteerism.

## Limitations and Conclusions

This study has a few limitations. First, the response rate is 36.6%. This is despite the fact that one of the authors is a volunteer with St. John Ambulance. The low response rate raises concerns of representativeness. The conclusions from this study must be derived keeping these factors in mind. However, this study has included volunteers from different parts of Malaysia. Second, our study looked at only one type of healthcare voluntary services, i.e., services related St. John Ambulance. In order to generalize it will be interesting to include other healthcare services such as hospitals. Third, this study used self-reported questionnaire to collect data that may cause common method variance. Although it is often considered preferable to have objective reports or supervisor reports or reports from peers, it is common that access to this type of data is simply not available in field research. Fourth, this study is cross-sectional and therefore difficult to establish causal relationships between variables [81].

Our study makes the following conclusions: (1) ISB-V has a direct effect on life satisfaction; (2) self-esteem of volunteers has impact on job performance (paid jobs) and life satisfaction of volunteers. Besides, it also has effect on frequency of volunteering (a dimension of ISB-V); (3) job performance of volunteers has an impact on their life satisfaction; and (4) current employment level of volunteers affects duration of volunteering, self-esteem, support from employers and job performance of volunteers. Even though this study was carried out in Malaysia among the volunteers of St. John Ambulance, we believe that the results can be useful to the healthcare volunteers and organizations that employ them in other parts of the world, especially in developing countries.
